# Biometrics based authentication scheme for session initiation protocol

**DOI:** 10.1186/s40064-016-2725-0

**Published:** 2016-07-11

**Authors:** Qi Xie, Zhixiong Tang

**Affiliations:** Key Laboratory of Cryptography and Network Security, Hangzhou Normal University, Hangzhou, 311121 China

**Keywords:** Authentication, Three-factor, Key agreement, Session initiation protocol

## Abstract

Many two-factor challenge-response based session initiation protocol (SIP) has been proposed, but most of them are vulnerable to smart card stolen attacks and password guessing attacks. In this paper, we propose a novel three-factor SIP authentication scheme using biometrics, password and smart card, and utilize the pi calculus-based formal verification tool ProVerif to prove that the proposed protocol achieves security and authentication. Furthermore, our protocol is highly efficient when compared to other related protocols.

## Background

The session initiation protocol (SIP) is an application layer controlling protocol for creation, modification and termination of Voice over Internet Protocol (VoIP) sessions with one or more participants. With the rapid growth of VoIP users, SIP is used in both the wireless and the wired networks widely. Originally, SIP authentication scheme is derived from HTTP digest authentication (Franks et al. 1999), which cannot resist server-spoofing attack and password guessing attack (Yang et al. 2005). Since then, various user authentication schemes for SIP have been proposed.

In 2005, Yang et al. (2005) proposed a new SIP authentication scheme based on Diffie-Hellman key exchange protocol, but Huang and Wei (2006) found that Yang et al.’s scheme has high computational costs and proposed an efficient SIP scheme. To improve the efficiency, Durlanik and Sogukpinar (2005) and Wu et al. (2009) also proposed SIP authentication protocols using the Elliptic Curve Cryptography (ECC), respectively. Unfortunately, Yang et al.’s and Huang et al.’s schemes suffer from the off-line password guessing attack (Jo et al. 2009), while Durlanik et al.’s and Wu et al.’s schemes are vulnerable to the Denning-Sacco attack and the off-line password guessing attack (Yoon et al. 2010b). Yoon et al. (2010b) presented an improved scheme to overcome these weaknesses. But Liu and Koenig pointed out that Yoon et al.’s SIP authentication scheme is still insecure against the off-line password guessing attack and the insider attack (Liu and Koenig 2011). Applying one-way hash function and the fast logic operations like exclusive-or, Tsai (2009) proposed a nonce based SIP authentication scheme. Later on, Yoon et al. (2010a) demonstrated that their scheme is vulnerable to Denning-Sacco attack, off-line password guessing attack and stolen-verifier attack, and proposed a new SIP authentication scheme. In 2012, Xie (2012) demonstrated that Yoon et al.’s scheme is still vulnerable to stolen-verifier attack and off-line password guessing attack, and proposed an improvement of Yoon et al.’s scheme, but Farash and Attari (2013) found that Xie’s protocol is also insecure against impersonation attack and off-line password guessing attack, and then they proposed an improved scheme to resolve these problems.

Recently, to enhance the performance and secrecy, Arshad and Ikram (2013) proposed an ECC-based SIP authentication protocol in 2013. But Tang and Liu (2013), He et al. (2012) and Pu et al. (2013) pointed out that Arshad et al.’s protocol is vulnerable to off-line password guessing attack. They also developed new schemes to enhance the security of Arshad et al.’s scheme. Later, Irshad et al. (2014) demonstrated that Tang et al.’s scheme cannot resist the server impersonation attack if an adversary can obtain the user’s password, and they proposed an improved protocol using ECC. Recently, Zhang et al. (2014) proposed a new password-based SIP authentication protocol, but Tu et al. (2015), Irshad et al. (2015) and Wu et al. (2013) showed that Zhang et al.’s protocol is vulnerable to the impersonation attack, and they proposed improved protocols respectively. After that, Arshad and Nikooghadam (2016) showed that Irshad et al.’s scheme is still vulnerable to impersonation attack. Farash (2016) and Mishra et al. (2016) found that Tu et al.’s protocol cannot resist the impersonation attack, and also presented improved schemes. It is worth mentioning that Mishra et al.’s scheme is a three-factor SIP authentication scheme, but it does not achieve perfect forward secrecy. Very recently, Chaudhry et al. (2015b) found that Tu et al.’s scheme is vulnerable to server impersonation attack. Moreover, both Tu et al.’s and Farash’s improved schemes cannot protect user’s privacy and suffer from replay and denial of services attacks. To enhance the security, they proposed a privacy preserving authentication scheme for SIP. Kumari et al. (2015) argued that Farash’s protocol cannot withstand impersonation attack, password guessing attack, and session-specific temporary information attack. Further, Kumari et al. proposed an improved protocol to fix the weaknesses of Farash’s protocol.

Many of above mentioned session initiation protocols are based on either password or both of password and smart card. However, password based protocol may suffer from password guessing attack, and smart card based protocol may suffer from smart card stolen attack by extracting information stored in smart card, even if the smart card is designed for achieving a certain level of tamper resistance (Witteman 2002). In order to solve password guessing attack and smart card stolen attack for SIP authentication scheme, we use user’s biometrics to protect user’s password and the sensitive information in smart card, since user’s biometrics have many advantages, such as it is difficult to be fabricated, distributed, lost, forgotten, guessed or copied (Li and Hwang 2010). On the other hand, fuzzy extractor can always output the same random string if the input biometrics has sufficient similarity to the stored biometrics (Dodis et al. 2004). Therefore, in this paper, we propose a biometrics-based SIP authentication scheme, and use pi calculus (Abadi and Fournet 2001) based formal verification tool ProVerif (Abadi et al. 2009) to prove authentication and security of the proposed protocol.

The rest of the paper is organized as follows. In “Biometrics-based SIP authentication scheme” section, we propose our Biometrics-based SIP authentication scheme. Security analysis and formal verification are given in “Security analysis and formal verification” section. “Security and performance comparisons” section compares the security and performance of our protocol to existing ones, and we conclude the paper in “Conclusions” section.

## Biometrics-based SIP authentication scheme

A biometrics based SIP authentication scheme is proposed in this section, which consists of three phases: registration, login and authentication, and password change. In this section, we first describe the construction of the fuzzy extractor, then we give the scheme specification of the proposed biometrics based SIP.

### Fuzzy extractor

Fuzzy extractor contains a pair of randomized procedures 〈“generate” (*Gen*), “reproduce” (*Rep*)〉. The procedure *Gen* is designed for inputting users’ biometrics BIO, and then outputting a random and uniform string $\eta$ as secret information as well as a random auxiliary string $\lambda$ as public information, namely, *Gen*(BIO) = ($\eta,\lambda$). The procedure *Rep* takes the biometrics $BIO^{*}$ and the auxiliary string $\lambda$ as inputs. Even if the inputted $BIO^{*}$ has slightly difference with BIO, as long as the difference is less than the threshold, the procedure *Rep* will generate the same string *η*, namely, *Rep*($BIO^{*}, \lambda)=\eta$. Though we cannot always get the same biometrics due to the impact of noisy data when sampling, fuzzy extractor can overcome this problem. Readers may refer to Dodis et al. (2004), Yang and Yang (2009) for the detailed introduction of fuzzy extractor. The notations used in this paper are given in Table 1.

**Table 1:** The notations

Notation	Description
$E$	An elliptic curve with large order $n$
$P$	A generator on *E* with large order $n$
$U_{i}$	The user $U_{i}$
$BIO_{i}$	The user $U_{i}$’s biometrics
$ID_{i}$	The user $U_{i}$’s identity
$pw_{i}$	The user $U_{i}$’s password
$S$	The server $S$
$x$	The server $S$’s secret key
$h()$	A secure one-way hash function
‖	A string concatenation operation
$\oplus$	A exclusive-or(XOR) operation

### Registration

A legal user $U_{i}$ must register in the remote server *S* beforehand by performing the following steps, as shown in Algorithm 1.

Step 1.: The user $U_{i}$ chooses a password $pw_{i}$, a random number $a_{i} \in Z_{n}^{*}$, computes $M = h(a_{i} \left\| {pw_{i}} \right.)$ and sends the register message $\left\{{ID_{i},M} \right\}$ to *S* via a secure channel.
Step 2.: After *S* receives the register request message $\left\{{ID_{i},M} \right\}$, *S* computes $R = M \oplus h(ID_{i} \left\| x\right.)$, stores *R* into a smart card and sends it to *U*_*i*_ through a secure channel.
Step 3.: After *U*_*i*_ obtains the smart card, he or she enters his or her biometrics $BIO_{i}$ on a specific device and computes *Gen*($BIO_{i})=(\eta,\lambda)$, $B = a_{i} \oplus h\left(\eta \right)$, $C = h(ID_{i} \left\| {pw_{i}} \right\|a_{i})$ and stores *B*, *C* and *λ* into the smart card. Thus, the smart card contains $\{B,C,\lambda,R\}$.

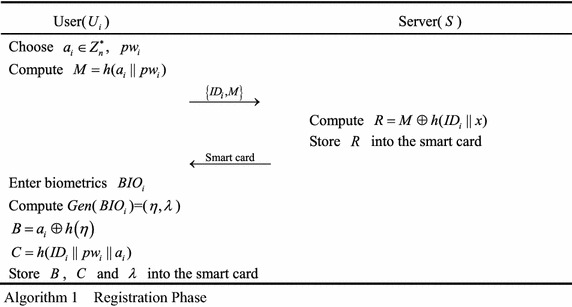


### Login and authentication

In this phase, *U*_*i*_ and *S* can be authenticated by each other and establish the session key. The process is shown in Algorithm 2.

Step 1.: The user $U_{i}$ inserts his or her smart card into a card reader, inputs his or her identity $ID_{i}$ and password $pw_{i}$, and enters biometrics $BIO_{i}^{*}$. The smart card selects a random number $b \in Z_{n}^{*}$, computes *Rep*($BIO_{i}^{*}$,$\lambda)\,=\,\eta$, $a_{i} = B \oplus h\left(\eta \right)$, and $C^{\prime} = h(ID_{i} \left\| {pw_{i}} \right\|a_{i})$. Then, the smart card checks whether $C^{\prime}$ is equal to *C*. If they are not equal, the protocol is terminated; otherwise, compute $D = bP$, $D^{\prime} = R \oplus h(a_{i} \| pw_{i}) = h(ID_{i} \|x)$ and $F = h(ID_{i} \left\|D\right\|D^{\prime})$. At last, $U_{i}$ sends the message $REQUEST\left\{{ID_{i},D,F} \right\}$ to *S*.
Step 2.: When the server *S* receives $REQUEST\left\{{ID_{i},D,F} \right\}$, *S* computes $D^{\prime\prime} = h(ID_{i} \left\| x \right.)$ and checks if $F$ and $h(ID_{i} \left\|D\right\|D^{\prime\prime})$ are equal. If they are not equal, *S* rejects the request; otherwise, *S* randomly chooses two numbers $u,t \in Z_{n}^{*}$, computes *H* = *uP*, $K = u \cdot h(ID_{i} \left\| {x)D} \right.$, $SK = h(ID_{i}\left\|t\right\|K)$ and $Auth_{s} = h(D\left\|K\right\|D^{\prime\prime}\left\|t\right\|SK\left\|H\right.)$. Finally, *S* sends the message $CHALLENGE\left\{{realm,Auth_{s},H,t} \right\}$ to *U*_*i*_.
Step 3.: When the user $U_{i}$ receives $CHALLENGE\left\{{realm,Auth_{s},H,t} \right\}$, he or she computes $K = bD^{\prime}H$ and $SK = h(ID_{i} \|t\|K)$. Then $U_{i}$ checks if $Auth_{s}$ and $h(D\|K\|D^{\prime}\|t\|SK\left\|H\right.)$ are equal. $U_{i}$ terminates the protocol if they are not equal; otherwise, $U_{i}$ computes $Auth_{u} = h(ID_{i} \left\| {realm} \right\|K\left\| {D^{\prime}} \right\|t\left\| {SK} \right\|H\left\| D \right.)$ and sends the message $RESPONSE\left\{{ID_{i},realm,Auth_{u}} \right\}$ to *S*.
Step 4.: When the server *S* receives $RESPONSE\left\{{ID_{i},realm,Auth_{u}} \right\}$, it checks whether $Auth_{u}$ is equal to $h(ID_{i} \left\| {realm} \right\|K\left\| {D^{\prime\prime}} \right\|t\left\| {SK} \right\|H\left\| D \right.)$. If so, *S* and $U_{i}$ established the session key *SK* .

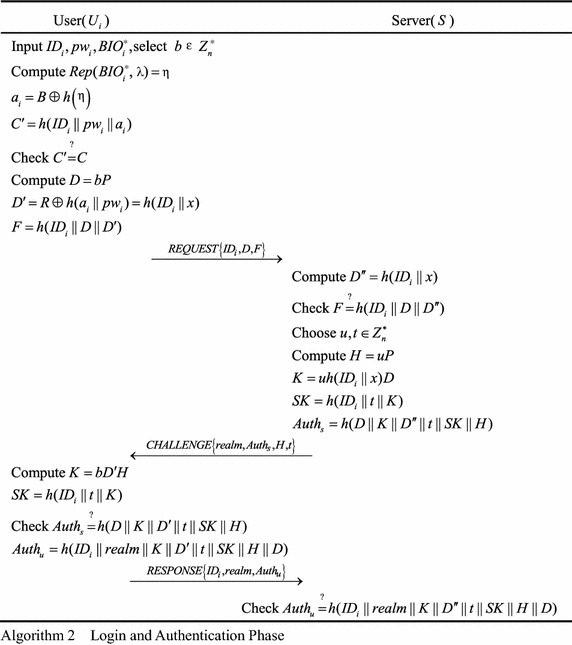


### Password change

The user $U_{i}$ inserts his or her smart card into a terminal, inputs his $ID_{i}$, old password $pw_{i}$, new password $pw_{i}^{new}$, chooses a random number $a_{i}^{new} \in Z_{n}^{*}$ and enters biometrics $BIO_{i}^{*}$ on a specific device. Then the smart card computes *Rep*($BIO_{i}^{*}$,$\lambda) = \eta$, $a_{i} = B \oplus h\left(\eta \right)$. After this, the smart card verifies $h(ID_{i} \left\| {pw_{i}} \right\|a_{i}) = C$. If it does not hold, the smart card rejects the request; otherwise, the smart card computes $R^{new} = h\left( {a_{i}^{new} \left\| {pw_{i}^{new} } \right.} \right) \oplus R \oplus h\left( {a_{i} \left\| {pw_{i} } \right.} \right)$, $B^{new} = a_{i}^{new} \oplus h\left(\eta \right)$ and $C^{new} = h(ID_{i} \left\| pw_{i}^{new} \right\|a_{i}^{new})$, and replaces $(R,B,C)$ with $(R^{new},B^{new},C^{new})$.

## Security analysis and formal verification

In this section, we will analyze the security of the proposed scheme.

### Formal verification

In order to prove the security of cryptographic protocols, there are some available formal verification tools, such as BAN logic (Burrows et al. 1989), AVISPA (Armando et al. 2005) and ProVerif. In this section, we prove secrecy and authentication using ProVerif, because it is performed automatically and efficiently, and can detect errors easily. ProVerif makes use of Dolev-Yao model (Dolev and Yao 1983) and supports many cryptographic primitives, including digital signature, symmetric and asymmetric encryption, hash function, and so on.

There’re two types of channels in the formal model: a public channel for transmitting general protocol messages and private channel for transmitting smart card data between user and his smart card. The definition of these channels is given as below:

- free cch: channel.
- free sch: channel [private].

The variables and constants used in the protocol are defined as follows:

- const P: bitstring.
- const BIO_i: bitstring.
- const pw_i: bitstring.
- const x: bitstring.
- free SK’: bitstring [private].
- free SK: bitstring [private].

The functions used in the protocol are defined as follows:

- fun sco(bitstring, bitstring): bitstring.
- fun Gen(bitstring): bitstring.
- fun Rep(bitstring, bitstring): bitstring.
- fun xor(bitstring, bitstring): bitstring.
- fun mult(bitstring, bitstring): bitstring.
- fun h(bitstring): bitstring.

Function sco, xor, mult, h represent bound symbol, exclusive or operation, scalar multiplication and hash function in the protocol, and function Gen and Rep are fuzzy extractor algorithms. The algebraic properties of these functions are modeled as the following equation and reduction:

- equation forall m: bitstring, n: bitstring; xor(xor(m, n), n) = m.
- In order to prove authentication, two events are defined as follows:
- event UserAuthed(bitstring).
- event UserStarted(bitstring).

The process part defines the action of participants and models the protocol as the parallel executions of them. According to the protocol, the following is the core message sequence for our protocol:

- Message 1: User $U_{i}$  → Server $S$: $REQUEST\left\{{ID_{i},D,F} \right\}$
- Message 2: Server $S$  → User $U_{i}$: $CHALLENGE\left\{{realm,Auth_{s},H,t} \right\}$
- Message 3: User $U_{i}$  → Server $S$: $RESPONSE\left\{{ID_{i},realm,Auth_{u}} \right\}$

The actions of user $U_{i}$ are composed of computing and then sending message 1 to *S*, waiting until he or she receives message 2 from *S*, computing and sending message 3 to *S*. We define user $U_{i}$ as below:
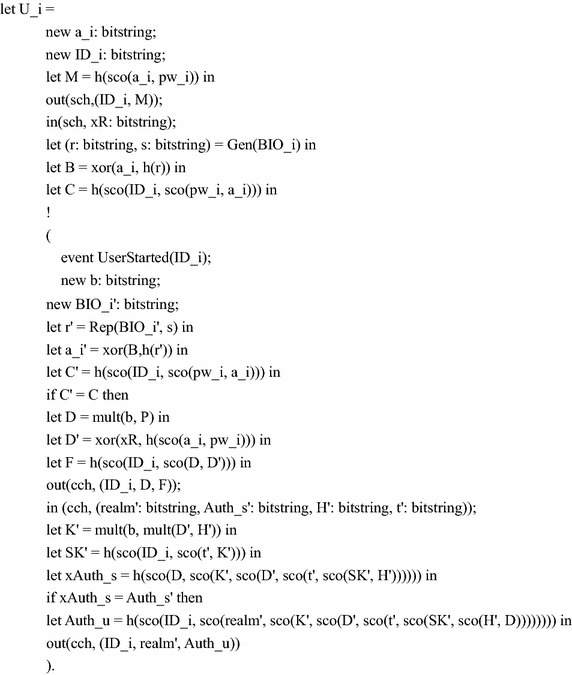


The actions of the server $S$ are composed of receiving message 1 from $U_{i}$, computing and sending message 2 to $U_{i}$, waiting until he receives message 3 from $U_{i}$, and then verifying the message 3. We define the server as below:
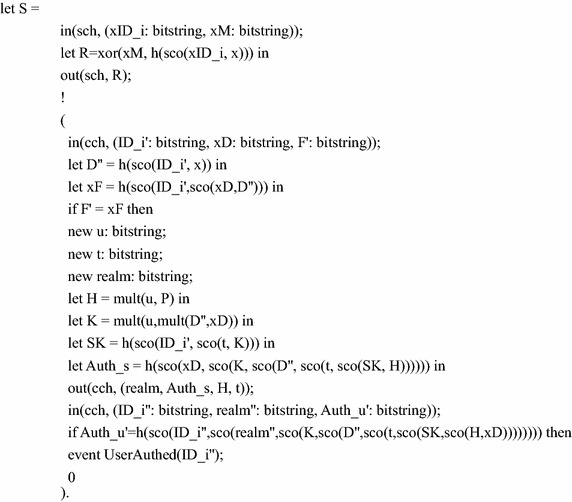


The protocol is defined as the parallel executions of the two participants:

- process !U_i| S

In order to verify mutual authentication and the session key security, we define the following queries for checking the events’ correspondence and the *attacker* queries respectively:

- query id: bitstring; inj-event(UserAuthed(id)) ==> inj-event(UserStarted(id)).
- query attacker(SK).
- query attacker(SK’).

**Fig. 1:**
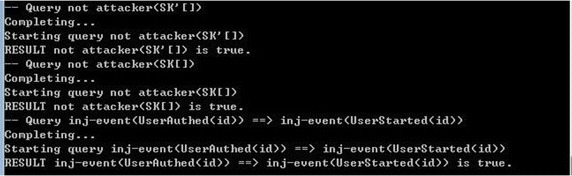
Verification result of the protocol

The above code is performed in the latest version 1.90 of ProVerif to show that the correspondence query is true and the two attacker queries are not true. That is, the authentication property and security are satisfied, referring to the Fig. 1.

### Security analysis

#### Session key security

Due to the impossibility of solving the computational Diffie-Hellman (CDH) problem, an adversary can neither know $h(ID_{i} \left\|x\right.)$ nor compute *ubP* from *bP* and *uP*. That is, the adversary cannot compute the session key $SK = h(ID_{i} \left\|t\right\|uh(ID_{i} \left\|x\right.)bP)$.

#### Mutual authentication

The user $U_{i}$ and the server *S* can authenticate each other by checking the correctness of *F*, $Auth_{u}$ and $Auth_{s}$, respectively. Without the knowledge of $h(ID_{i} \left\|x\right.)$, no one except the user and the server can compute $Auth_{u}$ and $Auth_{s}$.

#### Replay attack

An adversary may intercept the request message $REQUEST\left\{{ID_{i},D,F} \right\}$ and replay to the server, where $D = bP$, $D^{\prime} = h(ID_{i} \left\|x\right.)$ and $F = h(ID_{i} \left\|D\right\|D^{\prime})$. Without the knowledge of *b*, he or she cannot generate the correct response message $RESPONSE\left\{{ID_{i},realm,Auth_{u}} \right\}$ after receiving the server’s message $CHALLENGE\left\{{realm,Auth_{s},H,t} \right\}$. Then the server could detect the attack by checking the correctness of $Auth_{u}$. On the other hand, the adversary may intercept the challenge message $CHALLENGE\left\{{realm,Auth_{s},H,t} \right\}$ and replay it to the user, where $K = uh(ID_{i} \left\|x\right.)D$ and $Auth_{s} = h(D\left\|K\right\|D^{\prime\prime}\left\|t\right\|SK\left\|H\right.)$. As the user generates a new $D = bP$ for each session, the attack can be detected by checking the correctness of $Auth_{s}$. Therefore, proposed SIP authentication scheme can resist the replay attack.

#### Off-line password guessing attack

Suppose that the adversary gets the data $\left\{{B,C,\lambda,R} \right\}$, where $B = a_{i} \oplus h\left(\eta \right)$, $C = h(ID_{i} \left\|pw_{i} \right\|a_{i})$, $R = h(a_{i} \left\|pw_{i}) \oplus h(ID_{i} \right\|x)$. He could also eavesdrop the message $REQUEST\left\{{ID_{i},D,F} \right\}$, $CHALLENGE\left\{{realm,Auth_{s},H,t} \right\}$ and $RESPONSE\left\{{ID_{i},realm,Auth_{u}} \right\}$ transmitted between $U_{i}$ and *S*. The adversary may guess a password $pw_{i}^{*}$, but without the knowledge of *S*’s secret key *x*, he or she can neither compute the random number $a_{i}$ nor verify if his guessed password is correct or not. Hence, our scheme can resist the off-line password guessing attack.

For similar reasons, our protocol can resist smart card stolen attacks.

#### Privileged insider attack

In the registration phase of our scheme, $U_{i}$ chooses the random number $a_{i}$, the password $pw_{i}$, and computes the hash value $h(a_{i} \left\|pw_{i} \right.)$. Then $U_{i}$ sends the hash value to $S$. The privileged insider can’t get $pw_{i}$ as it is protected by the random number $a_{i}$ and the secure hash function.

#### Impersonation attack

Without the knowledge of $S$’s secret key $x$, the attacker can neither generate the valid challenge message $CHALLENGE\left\{{realm,Auth_{s},H,t} \right\}$, where $Auth_{s} = h(D\left\|K\right\|D^{\prime\prime}\left\|t\right\|SK\left\|H\right.)$ and $K = uh(ID_{i} \left\|x\right.)D$, nor compute the legal message $RESPONSE\left\{{ID_{i},realm,Auth_{u}} \right\}$. Note that all messages are transmitted via a secure channel in registration phase, which are supposed to be free of corruption. So our scheme could withstand the impersonation attack.

#### Stolen-verifier attack

In the proposed scheme, $S$ only needs to keep its key *x* secret. No password-verifier table is required to be stored in the server’s database. Therefore, our scheme can resist the stolen-verifier attack.

#### Man-in-the-middle attack

From the above security analysis, we know that our scheme could provide mutual authentication between $U_{i}$ and *S*, and can resist off-line password guessing attack and impersonation attack. Hence, our scheme is secure against the man-in-the-middle attack.

#### Perfect forward secrecy

In our protocol, the session key is $SK = h(ID_{i} \left\|t\right\|uh(ID_{i} \left\|x\right.)bP)$, even if an adversary corrupts all secret parameters such as *S*’s secret key *x* and $U_{i}$’s password $pw_{i}$, he or she still cannot compute $uh(ID_{i} \left\|x\right.)bP$ from $bP$ and $uP$ due to the intractability of CDH problem. Therefore, the introduced scheme can provide perfect forward secrecy.

## Security and performance comparisons

### Security and computation cost comparison

The security and computation cost comparisons between the proposed scheme and some related schemes (Zhang et al. 2014; Tu et al. 2015; Irshad et al. 2015; Arshad and Nikooghadam 2016; Farash 2016; Mishra et al. 2016; Chaudhry et al. 2015a; Wu et al. 2015) are given in Tables 2 and 3. For convenience, some notations are defined as follows: SY, H, MI, SM and PA are the operation times of a symmetric key encryption or decryption, hash function, modular inversion, scalar multiplication and point addition over elliptic curve, respectively.

**Table 2:** Security comparison

Schemes	Zhang et al. (2014)	Tu et al. (2015)	Irshad et al. (2015)	Arshad and Nikooghadam (2016)	Farash (2016)	Mishra et al. (2016)	Chaudhry et al. (2015a)	Wu et al. (2015)	Our scheme
Session key security	Y	Y	Y	Y	Y	Y	Y	Y	Y
Replay attack	Y	Y	Y	Y	Y	Y	Y	Y	Y
Perfect forward secrecy	Y	Y	Y	Y	Y	N	Y	Y	Y
Man-in-the-middle attack	Y	N	Y	Y	Y	Y	Y	Y	Y
Stolen-verifier attack	Y	Y	Y	Y	Y	Y	Y	Y	Y
Impersonation attack	N	N	N	N	N	Y	Y	Y	Y
Privileged insider attack	N	Y	Y	N	Y	Y	Y	N	Y
Mutual authentication	Y	Y	Y	Y	Y	Y	Y	Y	Y
Password guessing attack	Y	Y	Y	Y	N	Y	Y	Y	Y

*Y* the scheme can resist this attack or provide this property

*N* the scheme cannot resist this attack or cannot provide this property

**Table 3:** Computation cost comparison

Schemes	RP	LAAP	PCP	TC	AT (ms)
Zhang et al. (2014)	1SM + 2H	8SM + 2PA + 11H	1SM + 4SY + 6H	10SM + 2PA + 4SY + 19H	22.3797
Tu et al. (2015)	1SM + 2H	7SM + 1PA + 10H	1SM + 4SY + 6H	9SM + 1PA + 4SY + 18H	20.1226
Irshad et al. (2015)	1SM + 2H	7SM + 12H	1SM + 4SY + 6H	9SM + 4SY + 20H	20.0984
Arshad and Nikooghadam (2016)	2H	4SM + 8H + 1MI	9H	4SM + 19H + 1MI	8.9533
Farash (2016)	1SM + 2H	7SM + 1PA + 10H	1SM + 4SY + 6H	9SM + 1PA + 4SY + 18H	20.1226
Mishra et al. (2016)	4H	3SM + 12H	6H	3SM + 22H	7.184
Chaudhry et al. (2015a)	3H	6SM + 7H	3H	6SM + 13H	13.3859
Wu et al. (2015)	4H	4SM + 4SY + 12H	4H	4SM + 4SY + 20H	8.9684
Our scheme	4H	4SM + 12H	5H	4SM + 21H	8.9523

*RP* registration phase, *LAAP* login and authentication phase, *PCP* password change phase, *TC* total computation, *AT* actual time

Very recently, Kilinc and Yanik (2014) have estimated the complexity of various cryptographic operations by using the PBC library. The actual execution time for the above notations of operations are as follows: SY is about 0.0046 ms, H is about 0.0023 ms, MI is about 0.0056 ms (Koblitz et al. 2000), SM is about 2.226 ms, PA is about 0.0288 ms.

From Tables 2 and 3, we can conclude that our scheme enjoys better security than others, and higher efficiency than other related schemes except Mishra et al.’s protocol (Chaudhry et al. 2015a). Unfortunately, Mishra et al.’s protocol cannot provide perfect forward secrecy since the session key is

$$SK = h\left( {username\left\| {h(mk} \right\|username\left\| {N)} \right\||mk \cdot uP)_{x} \left\| {T_{2} } \right\|T_{3} } \right),$$

where $mk$ is the secret key of the server $S$, $T_{2}$ and $T_{3}$ are timestamps, $u$ is nonce chosen by the user and $N$ is registration sign. According to the definition of perfect forward secrecy, if an attacker can know the secret key $mk$ of $S$ then he or she can compute the session key $SK$. Generally, we can use Diffie-Hellman key exchange algorithm to achieve perfect forward secrecy, but it needs more scalar multiplication operations over elliptic curve.

### Storage capacity comparison

Since the proposed protocol is developed for applications using smart card, the memory requirement is a key parameter in concern. Therefore, we have also compared the storage capacity of our scheme with other related schemes (Zhang et al. 2014; Tu et al. 2015; Irshad et al. 2015; Arshad and Nikooghadam 2016; Farash 2016; Mishra et al. 2016; Chaudhry et al. 2015a; Wu et al. 2015). We assume that hash function outputs 256 bits, the size of a point on elliptic curve is 164 bits, the length of a random nonce is 128 bits, and the length of an identity is 128 bits. In the proposed scheme, the smart card needs to store $\{B,C,\lambda,R\}$ which is 256 + 256 + 128 + 256 = 896 bits. The storage capacities of other relevant schemes have been shown in Table 4, which shows that the memory of smart cards needed in all schemes are less than 1 k bit.

**Table 4:** Storage capacity comparison

Schemes	Zhang et al. (2014)	Tu et al. (2015)	Irshad et al. (2015)	Arshad and Nikooghadam (2016)	Farash (2016)	Mishra et al. (2016)	Chaudhry et al. (2015a)	Wu et al. (2015)	Our scheme
Memory needed in smart card (bits)	292	292	456	128	292	932	676	676	896

## Conclusions

In this paper, we propose a secure and efficient biometrics-based SIP authentication scheme. We apply formal verification tools and security analysis against various attacks to show that our proposed scheme achieves both security and authentication. Moreover, the performance evaluation validates that our scheme has very high efficiency in comparison to other related schemes.
